# The impact of plasma triglyceride and apolipoproteins concentrations on high-density lipoprotein subclasses distribution

**DOI:** 10.1186/1476-511X-10-17

**Published:** 2011-01-21

**Authors:** Li Tian, Yanhua Xu, Mingde Fu, Tao Peng, Yinghui Liu, Shiyin Long

**Affiliations:** 1 Laboratory of Endocrinology and Metabolism, West China Hospital, Sichuan University, Chengdu 610041, Sichuan, People's Republic of China; 2 Chengdu Hoist Biotechnology Co.,LTD, Chengdu 610075, Sichuan, PR China; 3 Chengdu University Affiliated Hospital of Traditional Chinese Medicine, Chengdu 610072, Sichuan, PR China; 4 Department of Biochemistry and Molecular Biology, University of South China, Hengyang, Hunan, People’s Republic of China

## Abstract

**Objective:**

To investigate the effect of triglyceride (TG) integrates with plasma major components of apolipoproteins in HDL subclasses distribution and further elicited the TG-apolipoproteins (apos) interaction in the processes of high density lipoprotein (HDL) mature metabolic and atherosclerosis related diseases.

**Methods:**

Contents of plasma HDL subclasses were quantities by two-dimensional gel electrophoresis associated with immunodetection in 500 Chinese subjects.

**Results:**

Contents of preβ_1_-HDL, HDL_3a_, and apoB-100 level along with apoB-100/A-I ratio were significantly increased, whereas there was a significant reduction in the contents of HDL_2_, apoA-I level as well as apoC-III/C-II ratio with increased TG concentration. Moreover, preβ_1_-HDL contents is elevated about 9 mg/L and HDL_2b _contents can be reduced 21 mg/L for 0.5 mmol/L increment in TG concentration. Moreover, with increase of apoA-I levels, HDL_2b _contents were marginally elevated in any TG concentration group. Furthermore, despite of in the apoB-100/A-I < 0.9 group, the contents of preβ_1_-HDL increased, and those of HDL_2b _decreased significantly for subjects in both high and very high TG levels compared to that in normal TG levels. Similarly, in the apoB-100/A-I ≥ 0.9 group, the distribution of HDL subclasses also showed abnormality for subjects with normal TG levels.

**Conclusions:**

The particle size of HDL subclasses tend to small with TG levels increased which indicated that HDL maturation might be impeded and efficiency of reverse cholesterol transport(RCT) might be weakened. These data suggest that TG levels were not only significantly associated with but liner with the contents of preβ_1_-HDL and HDL_2b_. They also raise the possibility that the TG levels effect on HDL maturation metabolism are subjected to plasma apolipoproteins and apolipoproteins ratios.

## Background

Several studies were published in the 1970s reporting an inverse association between plasma high density lipoprotein cholesterol (HDL-C) level and coronary heart disease (CHD) [1], confirmed since then by numerous additional studies throughout the world. HDL-C was first endorsed as a formal independent risk factor for CHD in the 1980s and has since evolved into one of the few "traditional" risk factors used by clinicians to assess cardiovascular risk. Because of the association of low HDL-C with other atherogenic factors, a low HDL-C is not as strongly independent in its prediction of CHD as suggested by usual multivariate analysis [2]. Subjects with low HDL-C display marked changes in their HDL composition and subclass distribution. Some studies indicate that larger HDL_2 _particles as well as HDL mean particle size are reduced in subjects with low HDL-C [3-7]. Case-control studies indicate that the inverse relation between HDL-C levels and CHD is accounted for by the largest HDL particles, the HDL_2b _subclass [8]. In addition, familial low-HDL-C subjects exhibit higher levels of inflammation markers such as high-sensitivity C-reactive protein (hsCRP) [9].

HDL metabolism is somewhat more complex than that of the other major lipoprotein fractions. It is well known that HDL does not represent a sum of identical particles but is rather comprised of discrete subclasses that differ related to charge, density, size, composition, shape and physiological functions [10]. Using two-dimensional gel electrophoresis coupled with immunoblotting, HDL can be divided into large, cholesterol-rich (HDL_2a _and HDL_2b_), small-sized (HDL_3c_, HDL_3b_, HDL_3a_, and preβ_1_-HDL) and preβ_2_-HDL [11,12]. Epidemiological studies have shown that individual HDL subclasses are not equally atheroprotective [13], a decrease content of the large-sized HDL_2b _particles and an increase content of the small-sized preβ_1_-HDL particles were highly and significantly associated with the risk of CHD[14,15].

Our laboratory had investigated the effect of plasma triglyceride (TG) and total cholesterol (TC) on the distribution of the HDL subclasses. The findings demonstrated that plasma increased TG and TC concentrations cause the particles size of HDL subclasses tend to smaller, creating an environment of diminished reverse cholesterol reverse cholesterol transport(RCT)[12,16-18]. The elevated TG levels are usually not only accompanied by other lipid metabolic disturbance but also associated with the change in plasma apolipoprotein levels. The TG and other lipids have been extensively studied for their effect on HDL subclasses distribution. Less information is known regarding the impact of TG combine with plasma apolipoproteins on profile of HDL subclasses distribution.

In this study, we mainly focused on the effect of TG integrate with plasma major components of apolipoproteins in HDL subclasses contents distribution and further elicited the TG-apolipoproteins interaction in the processes of HDL mature metabolism.

## Results

### Concentrations of plasma lipids, apolipoproteins, and ratios of lipids and apolipoproteins among subjects classified by levels of TG

Table 1 showed that in the population, the concentration of TG was 2.1 ± 0.3 mmol/L which exceeds normal TG levels recommended in the ATP-III of NCEP guidelines that indicated a substantial proportion of subjects with hypertriglyceridemia (HTG). Moreover, the body mass index(BMI) was increased with the elevation of the TG levels and the subjects in borderline-high, high and very high TG groups were characterized by an adverse lipid profile which comprised of increased concentrations of TG, TC, low density lipoprotein cholesterol(LDL-C) and levels of apoB-100, apoC-II, and apoC-III along with the ratios of TG/HDL-C, TC/HDL-C,LDL-C/HDL-C, and apoB-100/A-I; however, lower levels of HDL-C, apoA-I and apoC-III/C-II ratio compared with normal TG subjects.

**Table 1:** Concentrations of plasma lipids, apolipoproteins, and ratios of lipids, and apolipoproteins among subjects categorized by TG levels

	Total (n = 500)	Normal TG (n = 202)	Borderline-high TG (n = 77)	High TG (n = 183)	Very high TG (n = 38)
**Age(yr)**	56.7 ± 9.0	55.6 ± 9.7	55.8 ± 8.7	57.3 ± 9.2	58.6 ± 9.0
**Female/male**	175/325	72/130	26/51	64/119	13/25
**Sex ratio(%)**	53.8	55.4	50.9	53.8	52.0
**BMI(kg/m^2^)**	23.4 ± 2.2	22.4 ± 2.5	23.4 ± 2.8	24.5 ± 2.8^a§^	26.2 ± 3.0^a§ b§^
**TG(mmol/L)**	2.1 ± 0.3	1.0 ± 0.2	1.9 ± 0.2 ^a§^	3.3 ± 0.8^a§ b§^	7.1 ± 1.0^a§ b§ c§^
**TC(mmol/L)**	5.4 ± 0.9	5.0 ± 0.8	5.2 ± 0.9	5.5 ± 0.9^a‡^	6.1 ± 0.8^a§ b§ c‡^
**LDL-C(mmol/L)**	3.3 ± 0.4	2.8 ± 0.7	3.0 ± 0.8	3.4 ± 0.8^a‡b*^	3.5 ± 0.9^a‡b‡^
**HDL-C(mmol/L)**	1.2 ± 0.3	1.6 ± 0.3	1.3 ± 0.4^a§^	1.0 ± 0.4^a§b§^	0.9 ± 0.2^a§b§^
**ApoA-I(mg/L)**	1270.2 ± 102.5	1319.1 ± 193.8	1300.0 ± 178.4	1257.2 ± 151.2	1187.9 ± 123.2^a‡ b‡ c*^
**ApoB-100(mg/L)**	905.6 ± 98.4	804.3 ± 97.7	905.6 ± 108.4^a‡^	1015.4 ± 119.1^a§ b‡^	1167.2 ± 113.9^a§ b§ c‡^
**ApoC-II(mg/L)**	62.5 ± 10.8	45.4 ± 9.5	66.7 ± 11.9^a*^	101.9 ± 17.3^a§ b‡^	140.2 ± 25.3^a§ b§ c‡^
**ApoC-III(mg/L)**	150.2 ± 25.6	112.9 ± 21.9	152.3 ± 23.2^a§^	196.8 ± 27.5^a§ b§^	252.4 ± 29.8^a§ b§ c§^
**TG/HDL-C**	2.1 ± 0.2	0.8 ± 0.2	1.3 ± 0.3^a§^	3.5 ± 0.9^a§b§^	5.4 ± 0.9^a§ b§c§^
**TC/HDL-C**	4.8 ± 0.3	4.0 ± 1.0	4.7 ± 1.0^a*^	5.7 ± 1.0^a§b‡^	6.1 ± 1.1^a§ b§^
**LDL-C/HDL-C**	2.8 ± 0.2	1.3 ± 0.2	1.8 ± 0.3^a‡^	2.6 ± 0.5^a§b‡^	3.0 ± 0.9^a§ b§c*^
**ApoB-100/A-I**	0.7 ± 0.2	0.4 ± 0.1	0.5 ± 0.2	0.8 ± 0.2^a§b‡^	1.1 ± 0.3^a§ b§c‡^
**ApoC-III/C-II**	2.4 ± 0.3	2.5 ± 0.3	2.3 ± 0.2	1.9 ± 0.2^a§b‡^	1.8 ± 0.2^a§b‡^

Values are expressed as Mean ± S.D

*P< 0.05, ^‡^*P*< 0.01, ^§^*P*< 0.001

^a^Significantly different from Normal TG group

^b^Significantly different from Borderline-high TG group

^c^Significantly different from High TG group

### The apoA-I contents of HDL subclasses among subjects categorized based on levels of TG

Characteristics of the HDL subclass contents distribution profile were displayed in Table 2. The contents of small-sized particles preβ_1_-HDL, HDL_3a _were significantly increased, whereas there was a significant reduction in the contents of large-sized particles HDL_2a_, and HDL_2b _with the increased of TG levels. Furthermore, the contents of other HDL subclasses had no marked difference in borderline-high, high and very high TG subjects vs. the corresponding normal TG subjects.

**Table 2:** The apoA-I contents of HDL subclasses among subjects categorized by TG levels

	Normal TG (n = 202)	Borderline-high TG (n = 77)	High TG (n = 183)	Very high TG (n = 38)
**Preβ_1_-HDL(mg/L)**	80.6 ± 11.4	104.9 ± 17.8 ^a*^	129.5 ± 21.9^a§b‡^	153.0 ± 27.8^a§b‡ c*^
**Preβ_2_-HDL(mg/L)**	51.6 ± 8.6	58.9 ± 7.9	61.4 ± 8.7	64.9 ± 9.0
**HDL_3c_(mg/L)**	68.8 ± 9.2	71.4 ± 9.6	74.3 ± 9.0	75.8 ± 9.0
**HDL_3b_(mg/L)**	138.7 ± 20.7	143.7 ± 23.6	148.4 ± 21.9	151.4 ± 27.0
**HDL_3a_(mg/L)**	238.7 ± 30.6	285.5 ± 28.7^a‡^	310.9 ± 31.7^a§b*^	384.2 ± 40.1^a§b§c§^
**HDL_2a_(mg/L)**	292.7 ± 27.5	267.9 ± 24.1^a*^	227.5 ± 29.6^a§b‡^	202.4 ± 23.4^a§b§c*^
**HDL_2b_(mg/L)**	379.8 ± 57.8	319.3 ± 46.6^a§^	255.3 ± 27.3^a§b§^	219.3 ± 26.5^a§b§c‡^

Values are expressed as Mean ± S.D

*P< 0.05, ^‡^*P*< 0.01, ^§^*P*< 0.001

^a^Significantly different from Normal TG group

^b^Significantly different from Borderline-high TG group

^c^Significantly different from High TG group

### Relations of the preβ_1_-HDL and HDL_2b _contents to the levels of TG

**Figure 1:**
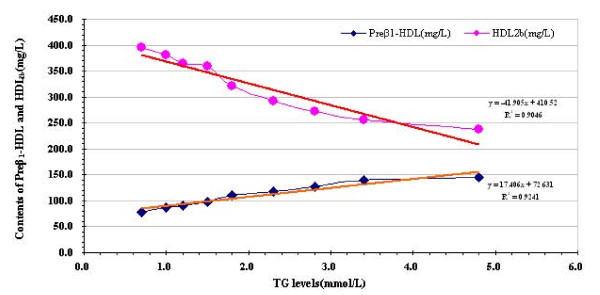
**The contents of preβ_1_-HDL and HDL_2b _according to approximately equal ninths of baseline TG for the entire study population**.

To investigate the degree of HDL subclasses change with the levels of TG varied, we divided the levels of TG into 9 strata and using each stratum TG median value as x axis, with every stratum TG corresponding the median preβ_1_-HDL and HDL_2b _contents as y axis to plot (Figure 1).The figure shows associations of TG levels with increased preβ_1_-HDL contents and with decreased HDL_2b _contents.

### Profile of preβ_1_-HDL and HDL_2b _contents distribution in accordance with the TG together with ApoA-I levels

**Figure 2:**
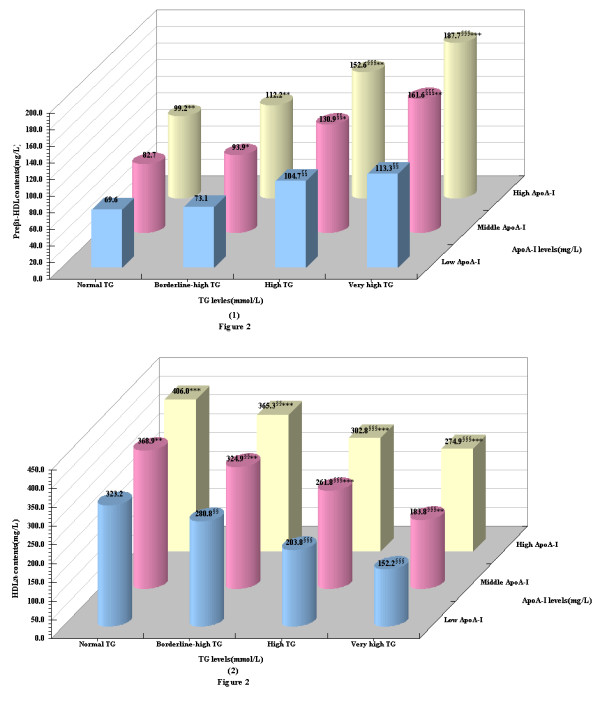
**The contents of preβ_1_-HDL(1) and HDL_2b_(2) according to TG and apoA-I concentration for the whole subjects**. *P< 0.05,**P< 0.01, ***P< 0.001, compared with the low apoA-I subgroup within the same TG group ^§§^*P*< 0.01, ^§§§^*P*< 0.001, compared with the normal TG subgroup within the same apoA-I group

Regardless of elevated TG and/or apoA-I, preβ_1_-HDL contents were obviously increased Figure 2(1); The similar results observed that in each same TG group, HDL_2b _contents were marginally elevated accompanied with the increase of apoA-I levels. On the contrary, in any apoA-I levels, contents of HDL_2b _were apparently decreased as the elevation of TG levels. Moreover, the lowest contents of HDL_2b _occurred in the very high TG-low apoA-I group (152.2 ± 23.8 mg/L) in terms of the graphic information represented in Figure 2(2).

### Changes of preβ_1_-HDL and HDL_2b _contents distribution based on the levels of TG and ApoB-100/A-I ratio

**Figure 3:**
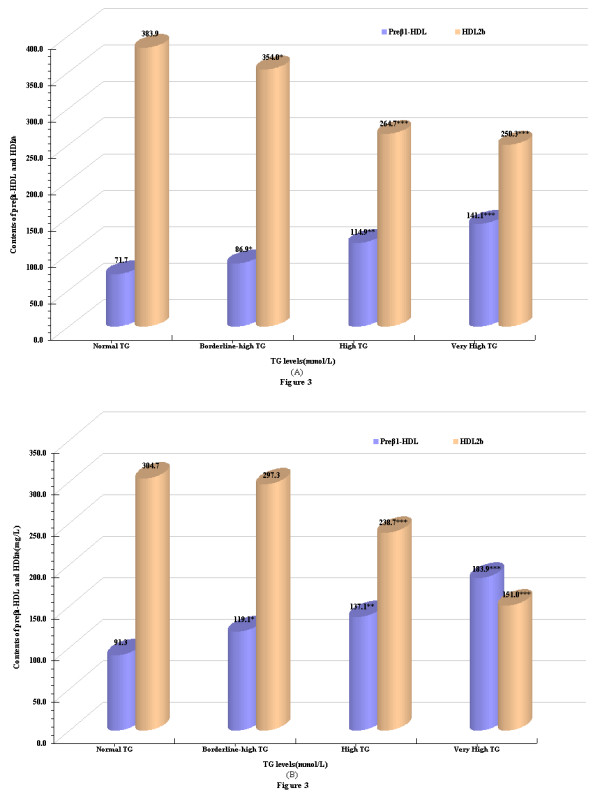
**The contents of preβ_1_-HDL and HDL_2b _for TG combine with apoB-100/A-I(A) and TG combine with apoB-100/A-I(B), respectively**. *P< 0.05,**P< 0.01, ***P< 0.001, compared with the normal TG subgroup

We use a suggested cut-point value of 0.9 for the apoB-100/apoA-I ratio to further dichotomized analyze the effect of TG concentration combined with ApoB-100/A-I ratio on profile of preβ_1_-HDL and HDL_2b _distribution [Figure 3(A) and 3(B)]. The results presented that both of apoB-100/A-I < 0.9 and apoB-100/A-I ≥ 0.9 groups, the contents of preβ_1_-HDL were increased significantly while the contents of HDL_2b _were obviously lower with a rise in TG concentrations.

It is noteworthy, the reduction of HDL_2b _contents (151.0 ± 21.3 mg/L) even exceed the elevation of preβ_1_-HDL contents(183.9 ± 28.7 mg/L) which resulted in a reduction of HDL_2b_/preβ_1_-HDL ratio (0.8) for subjects with very high TG-ApoB-100/A-I ≥0.9.

### The correlations analysis between plasma lipids, lipids ratios, and the contents of HDL subclasses after controlling for apoA-I, apoB-100

Table 3 shows correlations between the contents of HDL subclasses and conventional lipid parameter when controlling for apoA-I, and apoB-100. Specifically, the concentrations of TG, TC, LDL-C, and the ratios of TG/HDL-C, TC/HDL-C, as well as LDL-C/HDL-C were highly positively correlated with preβ_1_-HDL and HDL_3a _while being negatively associated with HDL-C concentration. The contents of HDL_2a _and HDL_2b _were inversely associated with TG and the ratios of TG/HDL-C, TC/HDL-C along with LDL-C/HDL-C but positively related to HDL-C concentration. With respect to other HDL subclasses, the concentrations of TG, TC, LDL-C, and the ratios of TC/HDL-C, LDL-C/HDL-C were positively correlated with HDL_3b_.

**Table 3:** Correlation coefficients between plasma lipids, lipids ratios and HDL subclasses contents (Controlling for ApoA-I, ApoB-100)

	TG		TC		LDL-C		HDL-C		TG/HDL-C		TC/HDL-C		LDL-C/HDL-C	
	t	*P*	t	*P*	t	*P*	t	*P*	t	*P*	t	*P*	t	*P*
**Preβ_1_-HDL**	.448	.000	.186	.000	.006	.897	-.269	.000	.439	.000	.374	.000	.152	.001
**Preβ_2_-HDL**	.147	.001	.029	.525	.047	.301	-.052	.254	.141	.001	.114	.012	.035	.446
**HDL_3c_**	.011	.209	.099	.028	.063	.164	.083	.067	.025	.587	.021	.643	.005	.912
**HDL_3b_**	.006	.899	.183	.000	.176	.000	-.030	.507	.005	.915	.116	.011	.147	.001
**HDL_3a_**	.288	.000	-.119	.009	-.176	.000	-.253	.000	.314	.000	.233	.000	.065	.156
**HDL_2a_**	-.211	.000	.059	.192	.097	.033	.207	.000	-.214	.000	-.164	.000	.049	.280
**HDL_2b_**	-.296	.000	-.146	.001	-.019	.674	.281	.000	-.314	.000	-.310	.000	-.165	.000

## Discussion

Many prospective studies have reported a positive relationship between levels of TG and incidence of CHD [19,20]. However; early multivariate analyses generally did not identify plasma TG as an independent risk factor for CHD [21]. Lipoprotein metabolism is integrally linked with, and elevations of plasma TG can be confounded by significant correlations with LDL and HDL-C levels. Nonlipid risk factors of obesity, cigarette smoking also integrated with TG as are several emerging risk factors [22]. Therefore, many persons with elevated TG are at increased risk for CHD, even when this greater risk can not be independently explained by TG.

It is well known that the most common hyperlipidemia for the Chinese population was characterized by elevated TG levels, which was much more prevalent (61% of total hyperlipidemia) than high TC [11]. Liu [23] suggested that higher levels of TG in China were induced by high-carbohydrate diets of the populations. Sugars and grains require insulin secretion, which is a potent stimulus to the liver to produce TG. We assessed the alteration of TG levels by Adult Treatment Panel III (ATP-III) guidelines [24] and HDL subclasses particle size using two-dimensional electrophoresis immunodetection. The data displayed that with the elevation of TG levels, the subjects have an atherogenic dyslipidemia phenotype comprising of an increased of TG, TC, LDL-C and apoB-100,C-II, C-III along with the ratios of TG/HDL-C, TC/HDL-C,LDL-C/HDL-C, and apoB-100/A-I, coupled with a decreased of HDL-C, apoA-I as well as apoC-III/C-II ratio. Meanwhile, elevated TG concentrations were related to the higher contents of small-sized (preβ_1_-HDL and HDL_3a_) and the lower contents of large-sized HDL (HDL_2a _and HDL_2b_). Further, for grouped analyses, individuals were classified according to approximately equal ninths of baseline TG for the entire study population (_0.7, 0.8-1.0, 1.1-1.4, 1.5-1.8, 1.9-2.3, 2.4-2.8, 2.9-3.4, 3.5-4.8, and ≥ 4.8 mmol/l). Trends in mean values of major HDL subclasses (preβ_1_-HDL and HDL_2b_) across these ninths were assessed through simple linear regression, in this models with the contents of preβ_1_-HDL and HDL_2b _as the dependent variable and the levels of TG levels as independent variable, our results revealed that preβ_1_-HDL contents is elevated about 9 mg/L and HDL_2b _contents can be reduced 21 mg/L for 0.5 mmol/L increment in TG.

Many studies have established that with the elevation of plasma TG concentration and the reduction of HDL-C concentration, cholesteryl ester transfer protein (CETP) and hepatic lipase (HL) activities were strengthen[25]. Through the actions CETP, intravascular exchange of neutral lipids and apos occurs between apoB-containing lipoprotein(LDL), intermediate density lipoprotein (IDL), very low density lipoprotein (VLDL), chylomicrons (CM), and remnants and HDL. Thus, elevated TG leads to the generation of TG-rich HDL particles, which are more susceptible to modification by HL [26]. HL has greater activity against HDL and converts larger HDL particles to smaller HDL particles. The higher concentration TG and the lower concentration of HDL-C associated with increased HDL catabolism, another is a reduction in lipoprotein lipase (LPL) and lecithin cholesterolacyl transferase (LCAT) activities. The activity of LCAT catalyzes the transfer of 2-acyl groups from lecithin to free cholesterol (FC), generating cholesterol ester (CE) and lysolecithin. Hydrophobic CE is retained in the HDL core forming larger mature HDL particles [27]. LPL has predominantly TG lipase activity, In the process of hydrolyzing TG-rich lipoprotein(TRL), CM and VLDL release TG, TC, phospholipids, apoA-I and apoCs, subsequent binding of these products to HDL_3 _results in formation of HDL_2 _particles. This modification leads to the formation of smaller HDL particles, which may impair its cardioprotective function.

Another important metabolic trigger for HDL mature metabolic disorder in the elevated TG concentrations condition could be involved in the plasma apolipoprotein levels alter. We divided the apoA-I concentrations into tertiles and to investigate the influence of TG combined with apoA-I levels on phenotype of HDL subclasses distribution. Our findings presented that regardless of elevated TG and/or apoA-I, preβ_1_-HDL contents were obviously increased; Likeness, in any TG levels, the contents of HDL_2b _significantly and gradually increased with elevated of apoA-I levels, which suggested that the protective effect of increased apoA-I levels against the reduction of HDL_2b _caused by elevated TG concentration. Other researchers have found that during severe sepsis or chronic inflammation, pro-inflammatory cytokine and TG levels are increased, while Apo-AI, Apo-B levels are decreased [28-31]. Cytokines have a major role in stimulating the production of acute phase proteins (APP) as part of the host immune response to infection, but prolonged inflammatory immune responses may be harmful to the host. Like albumin, Apo-AI is classified as a negative APP. While the role of negative APP is unclear, reduced levels of negative APP with anti-inflammatory activity, such as Apo-AI and transthyretin, may promote pro-inflammatory responses [32].

Studies in human apoA-I transgenic mice have showed that the distribution of apoA-I among the HDL subclasses was similar, suggesting that the content of apoA-I in the major HDL subspecies was fixed [33] and the apoA-I levels might reflect of the amount or number of HDL particles, and with the elevation of apoA-I levels, molecules of apoA-I distributed to each of the subclasses increased which resulted in all HDL subclasses tend to increase. In addition, apoA-I not only is activator of LCAT but also is a critical ligand of the HDL receptor scavenger receptor BI (SR-BI) and the interaction of apoA-I and SR-BI may facilitate hepatic selective uptake of HDL-C in the RCT pathway. In agreement with these, our previous study have reported that with increased plasma apoA-I concentration, the contents of all HDL subclasses rose significantly, with the increase in large-sized HDL_2b _being the most significant.

The large prospective apolipoprotein-related Mortality Risk (AMORIS) study [34] suggests that apoB-100, apoA-I and the apoB-100/apoA-I ratio should be regarded as highly predictive in evaluating cardiac risk [34,35]. A series of studies have shown that an apoB-100/apoA-I ratio ≥0.9 was a fair predictor of presence of the metabolic syndrome (MetS) [36], and men with an apoB-100/apoA-I ratio > 0.9 had also a faster growth of carotid artery intima-media thickness (IMT) than those below this value [37]. In the previous work, we use a 0.9 cut-point for the apoB-100/A-I ratio to dichotomized analyze the alteration of HDL subclasses distribution and found that preβ_1_-HDL contents were higher while HDL_2a_, HDL_2b _contents were lower for subjects with apoB-100/apoA-I ratio ≥0.9 than that for subjects with apoB-100/apoA-I ratio < 0.9, which suggests that apoB-100/A-I ratio might be a strong marker to HDL subclasses distribution, moreover, it could reflect sensitivity the alteration of HDL_2b_/preβ_1_-HDL values[38].

On the basis of this, we further discussed the effect of TG interact with apoB-100/apoA-I ratio ≥ 0.9 and apoB-100/apoA-I ratio < 0.9, separately on HDL subclasses distribution. The results obtained in the present work presented that, the preβ_1_-HDL contents increased significantly and the HDL_2b _contents decreased significantly as the elevation of TG concentration despite the subjects with the apoB-100/apoA-I ratio < 0.9. At the same time, in comparison with the normal TG, the marked lower values of HDL_2b_/preβ_1_-HDL in both high and very high TG groups (5.3 vs 2.3, 1.7). Similarly, in the apoB-100/apoA-I ratio ≥ 0.9, the HDL subclasses distribution might be reversed for subjects with normal TG concentration. A low HDL_2b_/preβ_1_-HDL ratio is a signature type of disturbed HDL metabolism and RCT and the cholesterol balance determined as the apoB-100/apoA-I ratio has repeatedly been shown to be a better index for risk assessment of CHD. The higher the value of the apoB-100/apoA-I ratio, the more cholesterol is circulating in the plasma compartment and this cholesterol is likely to be deposited in the arterial wall, provoking atherogenesis and risk of coronary vascular (CV) events. From the point of view of HDL subclasses distribution, all these findings revealed that when evaluation the CHD risk, relying only on apoB-100/apoA-I values for subjects might be inadequate and the concentration of TG should be concerned.

Besides, it is interesting to find that despite the levels of apoC-II and apoC-III increased, and an increasing in apoC-II more prominent than in apoC-III which conduces to the apoC-III/C-II ratio declined accompany with the elevation of TG concentration (Table 1). Sasaki, *et al *[39]. reported that high carbohydrate feedings caused decreased HDL-C and apoA-I concentrations were associated with reduced levels of apoC-III but increased concentrations of apoC-II in HDL_2_. In vitro investigation have exhibited that high levels of apoC-II have inhibited LPL activity rather than stimulated it [40]. The diets high in carbohydrate are prevalent in China, which may result in disorder of TG metabolism(excessive production and/or deficient clearance) Based on this, the high carbohydrate diets induced HTG usually accompanied with the apoC-III/C-II ratio decreasing, and the apoC-III/C-II ratio seems to play an important part in HDL subclasses maturation among Chinese population.

## Conclusions

In summary, we have shown that the particle size of HDL subclasses tend to small with increasing TG concentration which indicated that HDL maturation might be impeded and efficiency of RCT might be weakened. These data suggest that TG levels were not only significantly associated with but liner with the contents of preβ_1_-HDL and HDL_2b_. They also raise the possibility that TG levels effect on HDL maturation metabolism are subjected to plasma apos and apos ratios.

## Methods

### Subjects

Five hundred subjects (325 men; mean age, 56.3 ± 7.2 years; 175 women; mean age, 56.5 ± 8.1 years) were recruited to participate in a study examining plasma lipid and apo concentrations at West China Medical Center, Sichuan University. Exclusion criteria were the following: (1) the presence of nephrosis, diabetes mellitus, hypothyroidism, or hepatic impairment; (2) the presence of a major cardiovascular event (myocardial infarction, severe or unstable angina pectoris, and surgery) or stroke; (3) taking lipid-altering medications in the previous 1 month; or (4) a history of alcohol abuse and smoking cigarettes. Women were excluded if they had undergone a hysterectomy with or without an oophorectomy, or were postmenopausal and receiving hormone replacement therapy. Informed consent was obtained from each subject upon entry into the study population. This study protocol was approved by the ethics committee.

To study the TG role in the profile of HDL subclasses distribution, we divided these subjects into four subgroups according to plasma TG levels, that is normal (< 1.69 mmol/L), borderline-high (1.69-2.25 mmol/L), high (2.26-5.64 mmol/L), and very high (≥5.65 mmol/L) which were defined by following guidelines from the ATP-III of the National Cholesterol Education Program (NCEP) [24]. Moreover, the change in apoA-I, and apoB-100/A-I ratio associated with the phenotype of HDL subclass distribution were also investigated in the current work. The apoA-I concentration was entered as a categorical variable with three levels. Firstly, the apoA-I was arranged in ascending sequence on basis of its concentrations, next the number first to 167th subjects were designated as lowest tertile of apoA-I group; 168th to 334th subjects were designated as middle tertile of apoA-I group, and the 335th to 500th subjects were designated as the highest tertile of apoA-I group.

### Specimens

Whole blood specimens were drawn after a 12-hour overnight fast into EDTA-containing tubes. Plasma was separated within 1-2 hours. Plasma was stored at 4°C and used within 24 hour for lipid and apolipoprotein analyses. An aliquot of plasma was stored at -70°C for the determination of HDL subclasses.

### Plasma lipid and apolipoprotein analyses

Plasma TG, TC and HDL-C were measured by standard technique. TC and TG were determined with enzymatic kits (Beijing Zhongsheng Biotechnological Corporation, Beijing). HDL-C was determined after precipitation of the apoB-containing lipoproteins by phosphotungstate/magnesium chloride [41]. LDL-C was calculated using Friedwald formula (TG < 4.52 mmol/L) [42]. When plasma TG ≥ 4.52 mmol/L, LDL-C was determined following precipitation method with polyvinylsulfate (enzymatic kits). Plasma apoA-I, B-100 were determined by radial immunodiffusion methods [43] using kits developed at the apolipoprotein Research Laboratory, West China medical Center, Sichuan University. The intra-assay coefficient variations for apolipoprotein concentrations were between 2.1 and 4.8%, inter-assay coefficient of the variations was 3.5 to 7.9% [44].

### High-density lipoprotein cholesterol subclass analyses

ApoA-I-containing HDL subclasses were measured by nondenaturing two-dimensional gel electrophoresis associated with immunodetection method as described previously [45]. Briefly, 10 μl of plasma was first separated by charge on 0.7% agarose gel, into preβ and α mobility particles. In the second dimension, the 2 fractions of HDL were further separated according to size by 2-30% nondenaturing polyacrylamide gradient gel electrophoresis. To determine HDL subclasses, western blotting was conducted after electrophoresis, using horseradish peroxidase (HRP)-labeled goat anti-human apoA-I immunoglobulin G (IgG). HDL particle sizes were calibrated using a standard curve that includes bovine serum albumin, ferritin and thyroglobulin (Pharmacia Uppsala, Sweden). The calculation of each HDL subclass relative percentage (%) was based on the density of electrophoresis spots. Then the apoA-I contents (mg/L) of the HDL subclasses were calculated by multiplying the percentage of each subclass by the plasma total apoA-I concentrations. The inter-assay coefficient of variations of the relative concentration of preβ_1_-HDL, preβ_2_-HDL, HDL_3c_, HDL_3b_, HDL_3a_, HDL_2a_, and HDL_2b _in plasma sample were 9.4%, 9.8%, 4.9%, 6.2%, 7.3%, 11.1% and 7.9%, respectively (n = 5).

### Statistical analysis

All statistical analysis was performed using the statistical package SPSS Version 11.0 (SPSS Inc). Data are expressed as mean ± S.D. The significant differences between two groups were analyzed by one-way analysis of variance (ANOVA). Linear regression was used to examine the associations between plasma lipids, lipids ratios and other variables. In all comparisons, *P*< 0.05 (2-sided) was regarded as statistically significant.

## Conflict of Interest Statement

The authors declare that they have no conflict of interest.

## Authors' contributions

LT participated in the design of study and manuscript preparation along with editing. YHX conceived of the study, and helped to review the manuscript. MDF participated in manuscript reviewing and drafting. TP performed the data acquisition and analysis. YHL performed data and statistics analysis. SYL participated in drafted the manuscript. All authors read and approved the final manuscript.

## Notice of grant support

The authors thank the Fundamental Research Funds for the Central Universities (No. 2010SCU11029) for the grant support of this work.

## References

[B1] Miller GJ Miller NE Plasma-high-density-lipoprotein concentration and development of ischaemic heart-disease Lancet 1975 305 16 19 10.1016/S0140-6736(75)92376-4 46338

[B2] Vega GL Grundy SM Hypoalphalipoproteinemia (low high density lipoprotein) as a risk factor for coronary heart disease Curr Opin Lipidol 1996 7 209 216 10.1097/00041433-199608000-00007 8883496

[B3] Watanabe H Söderlund S Soro-Paavonen A Hiukka A Leinonen E Alagona C Salonen R Tuomainen TP Ehnholm C Jauhiainen M Taskinen MR Decreased high-density lipoprotein (HDL) particle size, preβ-, and large HDL subspecies concentration in Finnish low-HDL families: relationship with intima-media thickness Arterioscler Thromb Vasc Biol 2006 26 897 902 10.1161/01.ATV.0000209577.04246.c0 16469947

[B4] Nakanishi S Vikstedt R Söderlund S Lee-Rueckert M Hiukka A Ehnholm C Miulu M Metso J Naukkarinen J Palotie L Kovanen PT Jauhiainen M Taskinen MR Serum, but not monocyte macrophage foam cells derived from low HDL-C subjects, displays reduced cholesterol efflux capacity J Lipid Res 2009 50 183 192 10.1194/jlr.M800196-JLR200 18787236

[B5] Pascot A Lemieux I Prud'homme D Tremblay A Nadeau A Couillard C Bergeron J Lamarche B Després JP Reduced HDL particle size as an additional feature of the atherogenic dyslipidemia of abdominal obesity J Lipid Res 2001 42 2007 2014 11734573

[B6] Soro-Paavonen A Westerbacka J Ehnholm C Taskinen MR Metabolic syndrome aggravates the increased endothelial activation and low-grade inflammation in subjects with familial low HDL Ann Med 2006 38 229 238 10.1080/07853890500526352 16720437

[B7] Huang RC Mori TA Burke V Newnham J Stanley FJ Landau LI Kendall GE Oddy WH Beilin LJ Synergy between adiposity, insulin resistance, metabolic risk factors, and inflammation in adolescents Diabetes Care 2009 32 695 701 10.2337/dc08-1917 19131468 PMC2660473

[B8] Johansson J Carlson LA Landou C Hamsten A High density lipoprotein and coronary atherosclerosis. A strong inverse relation with the largest particles is confined to normotriglyceridemic patients Arterioscler Thromb Vasc Biol 1991 11 174 182 10.1161/01.atv.11.1.174 1987996

[B9] Shuhei N SÖderlund S jauhiainen M Taskinen MR Effect of HDL composition and particle size on the resistance of HDL to the oxidation Lipids in Health and Disease 2010 9 104 10.1186/1476-511X-9-104 20863394 PMC2954910

[B10] Von Eckardstein A Huang Y Assmann G Physiological role and clinical relevance of high-density lipoprotein subclasses Curr Opin Lipidol 1994 5 404 416 10.1097/00041433-199412000-00003 7712045

[B11] Wu XW Fu MD Liu BW Study on the immunodetection method of HDL subclasses in human serum Chin J Arterioscler 1999 7 253 255

[B12] Xu YH Fu MD Alterations of HDL subclasses in hyperlipidemia Clin Chim Acta 2003 332 95 102 10.1016/S0009-8981(03)00138-4 12763286

[B13] Kontush A Chapman MJ Antiatherogenic small, dense HDL-guardian angel of the arterial wall? Nat Clin Pract Cardiovasc Med 2006 3 144 153 10.1038/ncpcardio0500 16505860

[B14] Asztalos BF Cupples LA Demissie S Horvath KV Cox CE Batista MC Schaefer EJ High-density lipoprotein subpopulation profile and coronary heart disease prevalence in male participants of the Framingham Offspring Study Arterioscler Thromb Vasc Biol 2004 24 2181 2187 10.1161/01.ATV.0000146325.93749.a8 15388521

[B15] Cheung MC Brown BG Wolf AC Altered particle size distribution of apoA-I-containing HDL subpopulations in patients with coronary heart disease J lipid Res 1991 32 383 394 1906084

[B16] Yang YY Yan BY Fu MD Tian Y Relationship between plasma lipid concentrations and HDL subclasses Clin Chim Acta 2005 354 49 58 10.1016/j.cccn.2004.11.015 15748599

[B17] Gou LT Fu MD Xu YH Tian Y Yan BY Alterations of HDL subclasses in endogenous hypertriglyceridemia Am Heart J 2005 150 1039 1045 10.1016/j.ahj.2005.02.032 16290993

[B18] Jia LQ Fu MD Tian Y Xu YH Gou LT Tian HM Tian L Alterations of high-density lipoprotein subclasses in hypercholesterolemia and combined hyperlipidemia Int J Cardiol 2007 120 331 337 10.1016/j.ijcard.2006.10.007 17166608

[B19] Austin MA Hokanson JE Edwards KL Hypertriglyceridemia as a cardiovascular risk factor Am J Cardiol 1998 81 7B 12B 10.1016/S0002-9149(98)00031-9 9526807

[B20] Assmann G Schulte H Funke H Von Eckardstein A The emergence of triglycerides as a significant independent risk factor in coronary artery disease Eur Heart J 1998 19 M8 M14 9821011

[B21] Hulley SB Rosenman RH Bawol RD Brand RJ Epidemiology as a guide to clinical decisions:the association between triglyceride and coronary heart disease N Engl J Med 1980 302 1383 1389 10.1056/NEJM198006193022503 7374696

[B22] Grundy SM Hypertriglyceridemia, atherogenic dyslipidemia, and the metabolic syndrome Am J Cardiol 1998 81 18B 25B 10.1016/S0002-9149(98)00033-2 9526809

[B23] Liu BW Study on the pathogenesis of endogenous hypertriglyceridemia Chin J Arterioscler 1993 1 67 69

[B24] Executive Summary of The Third Report of The National Cholesterol Education Program (NCEP) Expert Panel On Detection, Evaluation, And Treatment of High Blood Cholesterol In Adults (Adult Treatment Panel III) JAMA 2001 285 2486 2497 10.1001/jama.285.19.2486 11368702

[B25] Rye KA Clay MA Barter PJ Remodeling of high density lipoproteins by plasma factors Atherosclerosis 1999 145 227 238 10.1016/S0021-9150(99)00150-1 10488948

[B26] Packard CJ Triacylglycerol-rich lipoproteins and the generation of small, dense low-density lipoprotein Biochem Soc Trans 2003 31 1066 1069 10.1042/BST0311066 14505481

[B27] Jonas A Lecithin cholesterol acyltransferase Biochim Biophys Acta 2000 1529 245 256 11111093 10.1016/s1388-1981(00)00153-0

[B28] Beers A Haas MJ Wong NC Mooradian AD Inhibition of apolipoprotein AI gene expression by tumor necrosis factor alpha: roles for MEK/ERK and JNK signaling Biochemistry 2006 45 2408 2413 10.1021/bi0518040 16475830

[B29] Alvarez C Ramos A Lipids, lipoproteins, and apoproteins in serum during infection Clin Chem 1986 32 142 145 3940695

[B30] Chenaud C Merlani PG Roux-Lombard P Burger D Harbarth S Luyasu S Graf JD Dayer JM Ricou B Low apolipoprotein A-I level at intensive care unit admission and systemic inflammatory response syndrome exacerbation Crit Care Med 2004 32 632 637 10.1097/01.CCM.0000114820.47460.0A 15090939

[B31] Khovidhunkit W Kim MS Memon RA Shigenaga JK Moser AH Feingold KR Grunfeld C Effects of infection and inflammation on lipid and lipoprotein metabolism: mechanisms and consequences to the host J Lipid Res 2004 45 1169 1196 10.1194/jlr.R300019-JLR200 15102878

[B32] Bresnihan B Gogarty M Fitzgerald O Dayer JM Burger D Apolipoprotein A-I infiltration in rheumatoid arthritis synovial tissue: a control mechanism of cytokine production? Arthritis Res Ther 2004 6 R563 R566 10.1186/ar1443 PMC1064871 15540281

[B33] Rubin EM Ishida BY Clift SM Kranss RM Expression of human apolipoprotein A-I in transgenic mice results in reduced plasma levels of murine apolipoprotein A-I and the appearance of two new high density lipoprotein size subclasses Biochemistry 1991 88 434 438 10.1073/pnas.88.2.434 PMC50825 1703299

[B34] Walldius G Jungner I Holme I Aastveit AH Kolar W Steiner E High apolipoproteinB, low apolipoproteinA-I, and improvement in the prediction of fatal myocardial infarction(AMORIS Study):a prospective study Lancet 2001 358 2026 2033 10.1016/S0140-6736(01)07098-2 11755609

[B35] Yusuf P Hawken S Ounpuu S Dans T Avezum A Lanas F McQueen M Budaj A Pais P Varigos J Lisheng L Effect of potentially modifiable risk factors associated with myocardial infarction in 52 countries (the INTER-GEART study):case-control study Lancet 2004 364 937 952 10.1016/S0140-6736(04)17018-9 15364185

[B36] Lind L Vessby B SundstrÖm J The apolipoproteinB/AI ratio and the metabolic syndrome independently predict risk for myocardial infarction in middle-aged men Arterioscler Thromb Vasc Biol 2006 26 406 410 10.1161/01.ATV.0000197827.12431.d0 16306426

[B37] Wallenfeldt K Bokemark L Wikstrand J Hulthe J Fagerberg B ApolipoproteinB/ApolipoproteinAI in relation to the metabolic syndrome and change in carotid artery intima-media thickness during 3 years in middle-aged men Stroke 2004 35 2248 2252 10.1161/01.STR.0000140629.65145.3c 15345795

[B38] Tian L Wu XW Fu MD Qin Y Xu YH Jia LQ Relationship between plasma apolipoproteinB concentrations, apolipoproteinB/apolipoproteinA-I and HDL subclasses distribution Clin Chim Acta 2008 388 148 155 10.1016/j.cca.2007.10.028 18036560

[B39] Sasaki N Holdworth G Barnhart RL Srivastava LS Glueck CS Kashyap ML Jackson RL Effect of a high carbohydrate diet on the content of apolipoproteinsCII, CIII and E in human plasma high density lipoprotein subfractions Atherosclerosis 1983 46 341 352 10.1016/0021-9150(83)90183-1 6847745

[B40] Havel RJ Fielding CJ Olivecrona T Shore VG Fielding PE Egelrud T Cofactor activity of protein components of human very low density lipoproteins in the hydrolysis of triglycerides by lipoprotein lipase from different sources Biochemistry 1973 12 1828 1833 10.1021/bi00733a026 4349259

[B41] Warnick GR Nguyen T Albers AA Comparison of improved precipitation methods for quantification of high-density lipoprotein cholesterol Clin Chem 1985 31 217 222 2578337

[B42] Friedewald WT Levy RI Fredrickson DS Estimation of the concentration of low-density lipoprotein cholesterol in plasma without the use of the preparative ultracentrifuge Clin Chem 1972 18 499 502 4337382

[B43] Labeur C Shepherd J Rosseneu M Immunological assays of apolipoproteins in plasma: methods and instrumentation Clin Chem 1990 36 591 597 2182220

[B44] Liu BW Wang KQ Immunoassay of human plasma apolipoproteins and clinical applications Lipoproteins and Atherosclerosis 1995 Beijing, People's Health Press 359 368

[B45] Fielding CJ Fielding PE Molecular physiology of reverse cholesterol transport J Lipid Res 1995 36 211 228 7751809

